# A model of factors influencing COVID-19 vaccine acceptance: A synthesis of the theory of reasoned action, conspiracy theory belief, awareness, perceived usefulness, and perceived ease of use

**DOI:** 10.1371/journal.pone.0261869

**Published:** 2022-01-12

**Authors:** Taslima Akther, Tasnima Nur

**Affiliations:** 1 Department of Accounting & Information Systems, Faculty of Business Studies, Jagannath University, Dhaka, Bangladesh; 2 Institute of Chartered Accountants of Bangladesh, Dhaka, Bangladesh; Univerza v Mariboru, SLOVENIA

## Abstract

The aim of this study is to investigate the key factors influencing the acceptance of COVID-19 vaccines and develop a model based on the theory of reasoned action, belief in conspiracy theory, awareness, perceived usefulness, and perceived ease of use. The authors created and distributed a self-administered online questionnaire using Google Forms. Data were collected from 351 respondents ranging in age from 19 to 30 years, studying at the graduate and postgraduate levels at various public universities in Bangladesh. The Partial Least Squares Structural Equation Modeling (PLS-SEM) method was used to analyze the data. The results indicate that belief in conspiracy theory undermines COVID-19 vaccine acceptance, thereby negatively impacting the individual attitudes, subjective norms, and acceptance. Individual awareness, on the other hand, has a strong positive influence on the COVID-19 vaccine acceptance. Furthermore, the perceived usefulness of vaccination and the perceived ease of obtaining the vaccine positively impact attitude and the acceptance of immunization. Individuals’ positive attitudes toward immunization and constructive subjective norms have a positive impact on vaccine acceptance. This study contributes to the literature by combining the theory of reasoned action with conspiracy theory, awareness, perceived usefulness, and perceived ease of use to understand vaccine acceptance behavior. Authorities should focus on campaigns that could reduce misinformation and conspiracy surrounding COVID-19 vaccination. The perceived usefulness of vaccination to prevent pandemics and continue normal education will lead to vaccination success. Furthermore, the ease with which people can obtain the vaccine and that it is free of cost will encourage students to get vaccinated to protect themselves, their families, and society.

## 1. Introduction

In December 2019, the first human cases of COVID-19, a coronavirus disease caused by SARS-CoV-2, were reported in Wuhan, China [1, 2]. The virus was initially called the novel or “New” coronavirus, but was later renamed SARS-CoV-2 by the International Committee on Taxonomy of Viruses, and the disease it causes was named “coronavirus disease 2019 or “COVID-19” by the World Health Organization (WHO) on February 11, 2020 [3]. Within a month, the novel coronavirus spread to 25 countries, including China and the WHO Director-General declared COVID-19 as the first pandemic caused by a coronavirus, causing over 118,000 infections in 114 countries and 4,291 deaths [4, 5]. After the initial infection in Wuhan, China, the first million COVID-19 cases were reported on April 2, 2020; the highest daily case count of 906,008 was on April 28, 2021. On May 18, 2021, over 4,500 people died in India, and over 4,400 people died in the US on January 12, 2021 [6]. The first COVID-19 patient was discovered in Bangladesh on March 8, 2020, and it was fatal on March 18, 2020. By June 2021, there were 840,000 cases, with 112 deaths in a single day on April 19, 2021. In Bangladesh, the Case Fatality Rate (CFR), which is the ratio of confirmed deaths to confirmed cases, increased by 58%, indicating that a greater proportion of infected people began to die [7].

During the public health emergency caused by COVID-19, the WHO placed unlicensed, still-in-development vaccines on the Emergency Use List as a temporary measure to make such vaccines available to those in need [8]. The WHO approved five vaccines for emergency use against COVID-19 as of June 3, 2021, because they met the necessary safety and efficacy criteria, including AstraZeneca/Oxford, Johnson and Johnson, Moderna, Pfizer/BioNTech, Sinopharm, and Sinovac [9]. Up until June 9, 2021, more than 944 million people received vaccine doses, and more than 480 million were fully vaccinated, representing 6.16 percent of the world’s population [6]. COVID-19 vaccines are expected to provide minimal protection against new virus variants while also preventing serious illness and death [10]. Rumors and conspiracy theories, on the other hand, contribute to COVID-19 vaccine hesitancy [11].

Conspiracy theories are primarily inferential beliefs derived from outside sources that extend beyond observable events [12]. Belief in conspiracy theories (BC) historically impeded population immunization. Previously, people refused immunizations due to false claims that vaccines contained infertility agents or spread infectious pathogens like the human immunodeficiency virus (HIV) [13]. People in many countries boycotted the polio vaccine due to rumors that it caused infertility, resulting in an increase in polio cases [13, 14]. Conspiracy theories about COVID-19 being a hoax or a bioweapon designed in a Chinese laboratory began to circulate on social media almost immediately after the first reports of the virus [15]. Bertin et al. [16] found that the more participants believed in COVID-19 conspiracy theories, the less likely they were to support vaccination. Sallam et al. [17] report a high prevalence of COVID-19 vaccine hesitancy among Jordanian university students, associated with conspiracy beliefs such as COVID-19 is a man-made disease, vaccination will be used to implant microchips into humans to control them, and vaccination can lead to infertility.

The theory of reasoned action (TRA) explains individual behavior by emphasizing the importance of beliefs in predicting behavior [18]. According to TRA, an individual’s attitude toward the outcome of the behavior and subjective norms (the opinions of the person’s social environment) predict individual behavior intention [19]. Positive vaccination attitudes will increase the rate of COVID-19 vaccine acceptance. Those who intend to receive the COVID-19 see high perceived benefits in doing so for the purpose of protecting themselves and others in their circle, implying vaccination compliance [20]. Raising public and individual awareness is the most important factor in the fight against diseases, crime, and social injustice [21, 22]. People are either unaware of or fearful of the current vaccination program [23].

This study investigates the factors that influence COVID-19 vaccine acceptance among the young generations of Bangladeshi public university students. Bangladesh, a developing country in South Asia, accounts for 0.58% of the world’s COVID-19 cases and is in the top 26 countries worldwide.. As a tertiary educational institute, public universities provide higher education, and the pandemic wreaked havoc on the students’ education and future prospects. At the height of the pandemic, we assessed the acceptance of COVID-19 vaccines among students at various public universities in Bangladesh, for whom vaccination is a critical issue in allowing them to resume their education and prepare them for their profession. We construct a model of COVID-19 vaccine acceptance using the frames of TRA, belief in conspiracy theory (BC), awareness (AW), perceived usefulness (PU), and perceived ease of use (PE). Our findings are a valuable resource for understanding vaccine acceptance behavior during this critical pandemic period and providing policymakers with some practical recommendations. Furthermore, this study makes a significant theoretical contribution to the literature by decomposing TRA using BC, AW, PU, and PE.

The remainder of the paper proceeds as follows. Section 2 discusses the COVID-19 pandemic and vaccination in the context of Bangladesh. Section 3 describes the theoretical framework and hypothesis development, followed by a description of the data and methodology in Section 4. Section 5 reports the analysis and results, and Section 6 provides a discussion, the implications of the study, the limitations, and conclusion.

## 2. The COVID-19 pandemic in Bangladesh and the vaccination context

**Fig 1:**
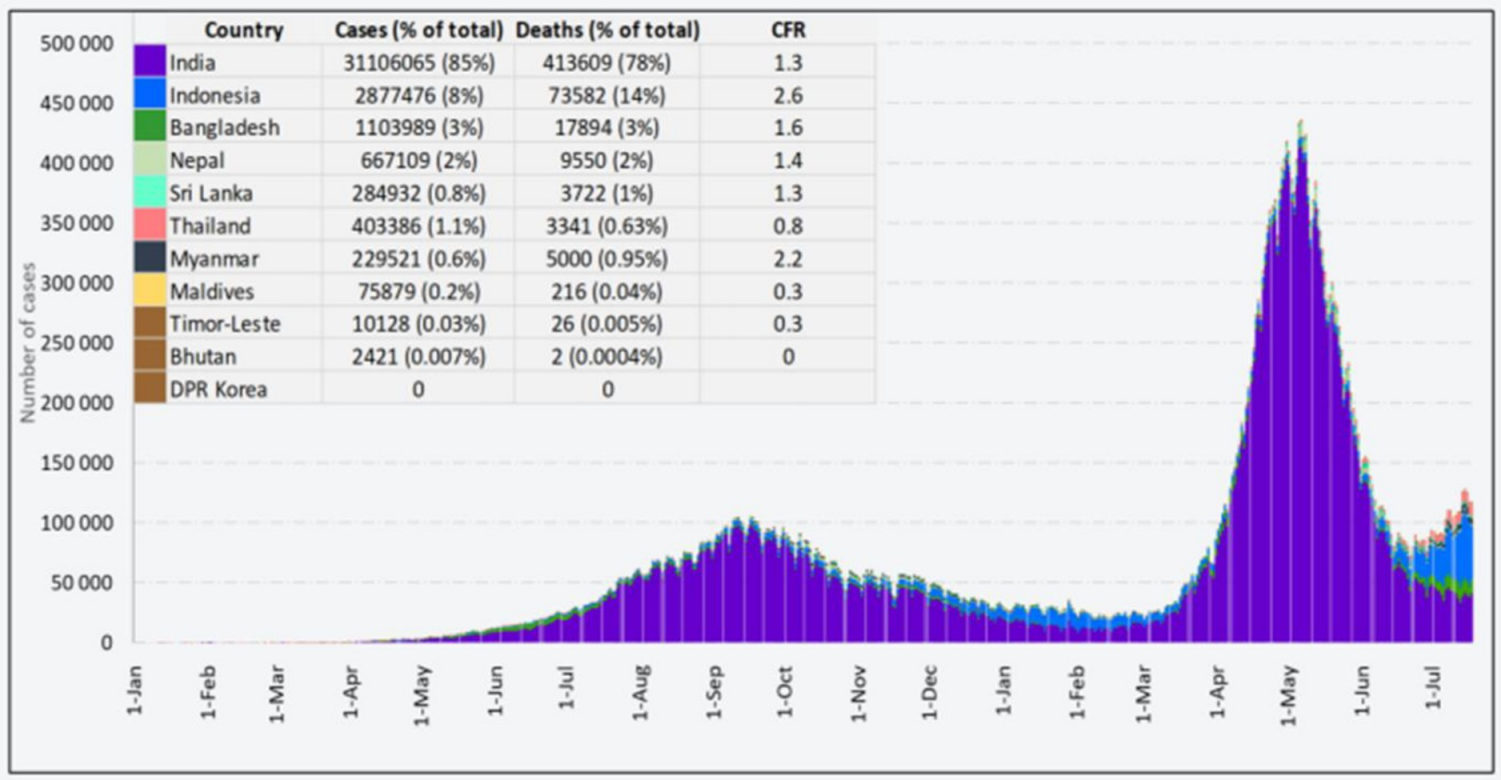
Cumulative reported number of COVID-19 cases, deaths, and transmission classification in SEAR countries.

Till June 2021, Bangladesh had 840,000 cases and over 13,000 deaths due to COVID-19. Prothomalo [24] published news of a study by the International Centre for Diarrhoea Disease Research, Bangladesh (ICDDR,B) showing that 71% and 55% residents of Dhaka and Chittagong, respectively, developed COVID-19 antibodies between October 2020 and February 2021 and many of these people were asymptomatic and so more dangerous to others in terms of spreading the virus unknowingly. Data reported by WHO in the following Fig 1 depicts that among the Southeast nations, Bangladesh has an upward trend in active cases, deaths, and the CFR rate from 1 January 2020 to 18 July 2021.

COVID-19 primarily infected young professionals, students, and working people in Bangladesh. According to the IEDCR, 68% of COVID-19 cases were observed in people aged 21 to 50 years, whereas infected patients aged >50 years made up 21% of total infected people, and children and youths aged 20 years made up were 11% of total infected cases [25]. Aside from direct consequences such as infection and death, COVID-19 affected the economic and social lives of Bangladeshis due to the nationwide lockdowns beginning March 24, 2020. The COVID-19 pandemic also highlighted flaws in Bangladesh’s healthcare system. As healthcare workers work to save the lives of COVID-19 patients, regular healthcare services and vaccination programs are hampered [26, 27]. On February 7, 2021, mass vaccination against COVID-19 began across the country based on online registration. Bangladesh has 53 government-funded public universities that operate as self-governing organizations. These universities are classified into agricultural universities, science and technology universities, engineering universities, medical universities, and general studies universities [28]. Due to the COVID-19 pandemic outbreak in Bangladesh, all educational institutions have been closed since March 2020. Many of these students also live in rented houses, as residential halls are also closed, which increases their expenses or reduces their savings. Due to a lack of digital learning opportunities in rural areas, education for a whole generation was disrupted.

## 3. Theoretical framework and hypothesis development

### 3.1. Belief in conspiracy theories and COVID-19 vaccination

Conspiracy theories are explanations for significant events that involve secret plots by powerful malevolent groups [29]. Commonly accepted conspiracy theories contend that climate change is a hoax [30]; NASA faked the moon landings [31]; the US government orchestrated the 9/11 terrorist attacks [32]; and vaccinations are harmful, but this fact is concealed to maintain profits [33]. A conspiracy belief is the unwarranted assumption of a conspiracy when other explanations are more likely [34]. A growing body of research shows that BC can have negative consequences on attitudes and behavior [35]. In addition to the social and political domains, BC has a significant impact on health. Conspiracy beliefs about the origin and treatment of HIV/AIDS had a negative impact on attitudes toward preventative measures and adherence to treatment programs [36]. Fears about the safety of childhood vaccinations contributed to a drop in polio vaccination rates in some countries [37].

### 3.2. Impact of conspiracy beliefs on attitude toward vaccination

Although vaccines are the most effective way to prevent infectious diseases, their safety and efficacy have long been the subject of conspiracy theories, with the central argument being that large pharmaceutical companies and/or governments conceal vaccine information for personal gain [37, 38]. Belief in anti-vaccine conspiracy theories reduces vaccination intentions [39]. Rumors about COVID-19 vaccine development delays, or that vaccines will be freely available only to supporters of the ruling government, may foster distrust between government stakeholders and the general public. This could affect the implementation of any vaccine-related policy. Online health information is frequently amplified by rumors and conspiracy theories that are not always based on scientific evidence [40]. Users who seek health information on online platforms are at risk of exposure to misinformation that could endanger public health [41]. Stecula et al. [42] discovered that people exposed to COVID-19 vaccine-related information on social media were more likely to be misinformed and vaccine-hesitant. We therefore hypothesize that

**H1: BC has a negative impact on attitude toward COVID-19 vaccine acceptance**.

### 3.3. Impact of conspiracy beliefs on subjective norms

Conspiracy beliefs are widespread and can have negative consequences because perceived social norms have a strong influence on individuals [43]. Social influence is the process by which perceptions of what other people think and do influence beliefs and behaviors [44]. Social norms guide behavior by implicitly defining what is and is not acceptable in a given context [44]. Sherif [45] defined social norms as mutually agreed-upon rules for social behavior. People who identify more strongly with the group are more likely to act in accordance with group norms [46]. Thus, perceived norms of conspiracy belief may influence personal BC, especially if people perceive groups with which they identify strongly as endorsing conspiracy theories [46, 47].

BC, particularly anti-vaccine conspiracy theories, is regarded as more normative than it is [43]. This is significant because the overestimation of in-group conspiracy beliefs may result in unwarranted social pressure to also endorse conspiracy beliefs given the negative social and health consequences of harboring conspiracy beliefs, particularly anti-vaccine conspiracy theories [30, 48]. It is concerning that perceived social norms may be driving conspiracy belief. We thus hypothesize

**H2: BC has a negative impact on subjective norms regarding COVID-19 vaccine acceptance**.

### 3. 4. Impact of conspiracy beliefs on COVID-19 vaccine acceptance

Rumors and conspiracy theories can contribute to vaccine apprehension [49]. Negative claims about vaccine effectiveness historically influenced vaccine uptake. Rumors about vaccination campaigns being used for political purposes are not new, and such rumors affected vaccination campaigns in some countries [50]. One prevalent rumor holds that critical phases of clinical trials in the vaccine development were skipped because pharmaceutical companies would not compensate participants for adverse side effects experienced during the trial. The most widely circulated rumor is that the COVID-19 vaccine would be a messenger Ribonucleic acid (mRNA) vaccine that could change people’s Deoxyribonucleic acid (DNA), transforming them into genetically modified humans, or cause cancers and infertility. Some claims emphasize that the COVID-19 vaccine was intended to reduce the global population. In Bangladesh, there was a rumor that China wanted to use Bangladeshi citizens as genuine pigs for a vaccine trial [49]. Therefore, we hypothesize

**H3: BC has a negative impact on COVID-19 vaccine acceptance**.

### 3. 5. Awareness

Awareness is the extent to which a target population is aware of an innovation and formed a general perception of what it entails [51]. The concept of awareness first appeared in innovation diffusion theory, which states that the decision-making process for adopting new technologies includes awareness, attitude formation, decision, implementation, and confirmation [52]. It is further defined as an individual’s active participation and increased interest in focal issues [53, 54]. The concept of awareness is central to human behavior in the social science, criminal justice, and medical behavioral science literature [21]. Awareness is one of the most important components of consciousness-raising because it fosters an understanding of the needs, impetus, and specificity of issues, events, and processes, and it is positively related to individuals’ attitudes and cognitive development [22, 55].

One study shows that people are either unaware of or fearful of the current vaccination program [23]. Vaccine hesitancy and misinformation are major impediments to achieving coverage and population immunization in many countries [56, 57]. Awareness can have positive impact on the vaccine acceptance. Thus, we propose

**H4: Awareness positively influences attitudes toward COVID-19 vaccine acceptance**.

In addition to an individual’s attitude toward behavior, the behavioral norms of an individual user’s social group have a strong influence on the individual’s behavioral intention [58]. The process of increasing problem awareness guides the development of a social network of organizations that strongly advocate for policies and programs to reduce such problems [59]. In the case of the COVID-19 vaccine, raising awareness among various social groups and communities raises community norms about COVID-19 and influences immunization through the COVID-19 vaccine. We therefore hypothesize that

**H5: Awareness positively influences subjective norms about taking the COVID-19 vaccine**.

Awareness is critical to technology acceptance. Identity theft, negative publicity, significant financial loss, and uncertain legal consequences could be devastating to individuals and organizations if they do not adopt protective technologies. Because such consequences are frequently reported in the popular media, we contend that awareness alone can motivate a user to act, regardless of whether he or she formed a positive attitude or is influenced by social group norms. Other studies on crime and disease prevention show that increased awareness has a direct influence on the intention to engage in certain behaviors [60, 61]. Consequently, we postulate that

**H6: Awareness has a positive impact on COVID-19 vaccine acceptance**.

### 3. 6. Perceived usefulness (PU) and perceived ease of use (PE)

#### 3. 6. 1. Perceived usefulness (PU)

PU is peoples’ subjective assessments of the extent to which using a system would improve their job performance [62]. It is the magnitude to which an individual considers using something that provides more benefits [63]. PU is a predictor of attitude [62, 64–66]. Users can develop a positive attitude because engaging in a particular behavior has numerous benefits. Islam et al. [67] demonstrated that the majority of participants had a positive attitude toward vaccination for its usefulness in protecting against COVID-19 disease. Perceived benefits, such as the COVID-19 vaccine’s high effectiveness in preventing significant suffering and complications of the disease, as well as the risk of becoming infected or infecting others, can predict COVID-19 vaccine acceptance. For recent graduates, a vaccine certificate may be required for new job applications, higher education applications, and proper continuation of their current studies and life activities. The usefulness of the vaccine creates a favorable attitude toward the acceptance of vaccines and the COVID-19 vaccine. Thus, we hypothesize that

**H7: The PU of the COVID-19 vaccine is positively related to attitude toward its acceptance**.

**H8: The PU of the COVID-19 vaccine has a positive impact on its acceptance**.

#### 3. 6. 2. Perceived ease of use (PE)

PE is an individual’s expectation of how easy the target system will be to understand, learn, and use [62, 63]. The complexity of a single system will impede the adoption of an innovation [52]. With less complexity in a system’s operation, a user can develop a positive attitude toward intention and behavior. PE has a direct relationship with attitude and acceptance [62, 66, 68]. Vaccination convenience is the ease of obtaining the vaccine, including factors such as physical availability, affordability, and accessibility [69]. When investigating vaccine acceptability, it is critical to consider vaccine convenience in terms of availability and affordability. If the COVID-19 vaccine can be obtained with less effort and is freely available, its acceptance will increase. Therefore, we propose

**H9: The PE of the COVID-19 vaccine is positively related to its acceptance**.

**H10: The PE of the COVID-19 vaccine is positively related to its acceptance**.

### 3. 7. Attitude, subjective norms, and COVID-19 vaccine acceptance

The theory of reasoned action, TRA [19], developed in the field of Social Psychology, has been widely used to explain individual behavior. The TRA hypothesizes that an individual’s intention to engage in a given behavior predicts behavior. In turn, subjective norms, that is, the individual’s attitude toward the outcome of the behavior and the opinions of the person’s social environment predict intention [19]. TRA is a general structure designed to explain almost all human behavior based on the significance of an individual’s beliefs in predicting their behavior [19, 70].

Vaccines are effective interventions that can help to reduce the global disease burden. Public vaccine hesitancy, on the other hand, is a pressing issue for public health officials. With the availability of COVID-19 vaccines, there is little information available on public acceptability and attitudes toward the COVID-19 vaccines. A positive attitude toward the vaccination will help to prevent COVID-19 and impact vaccinate acceptance. In a survey conducted across 19 countries, 71.5% respondents stated that they would take a vaccine if it were proven safe and effective [71]. We anticipate that positive attitudes toward vaccination will increase the rate of COVID-19 vaccine acceptance.

**H11: COVID-19 vaccine acceptance is related to attitude**.

Subjective norms are an individual’s perception of the social pressure to perform or refrain from performing a target behavior [19]. Normative beliefs reflect an individual’s perception of the influence of opinion among reference groups, whereas motivation to comply reflects the extent to which the individual wishes to comply with the wishes of the referent other [72]. Hence, people frequently act based on their perception of what others think they should do, and people close to them may influence their intention to adopt a behavior.

Subjective norms and self-efficacy are significant predictors of COVID-19 vaccination intention [73]. Subjective norms that particularly influenced respondents were when friends and family members responded positively to the vaccination. Individuals with a constructive outlook regarding COVID-19 vaccines would recommend it to their friends, family and the community. Hence, we postulate that

**H12: COVID-19 vaccine acceptance is related to subjective norms**.

**Fig 2:**
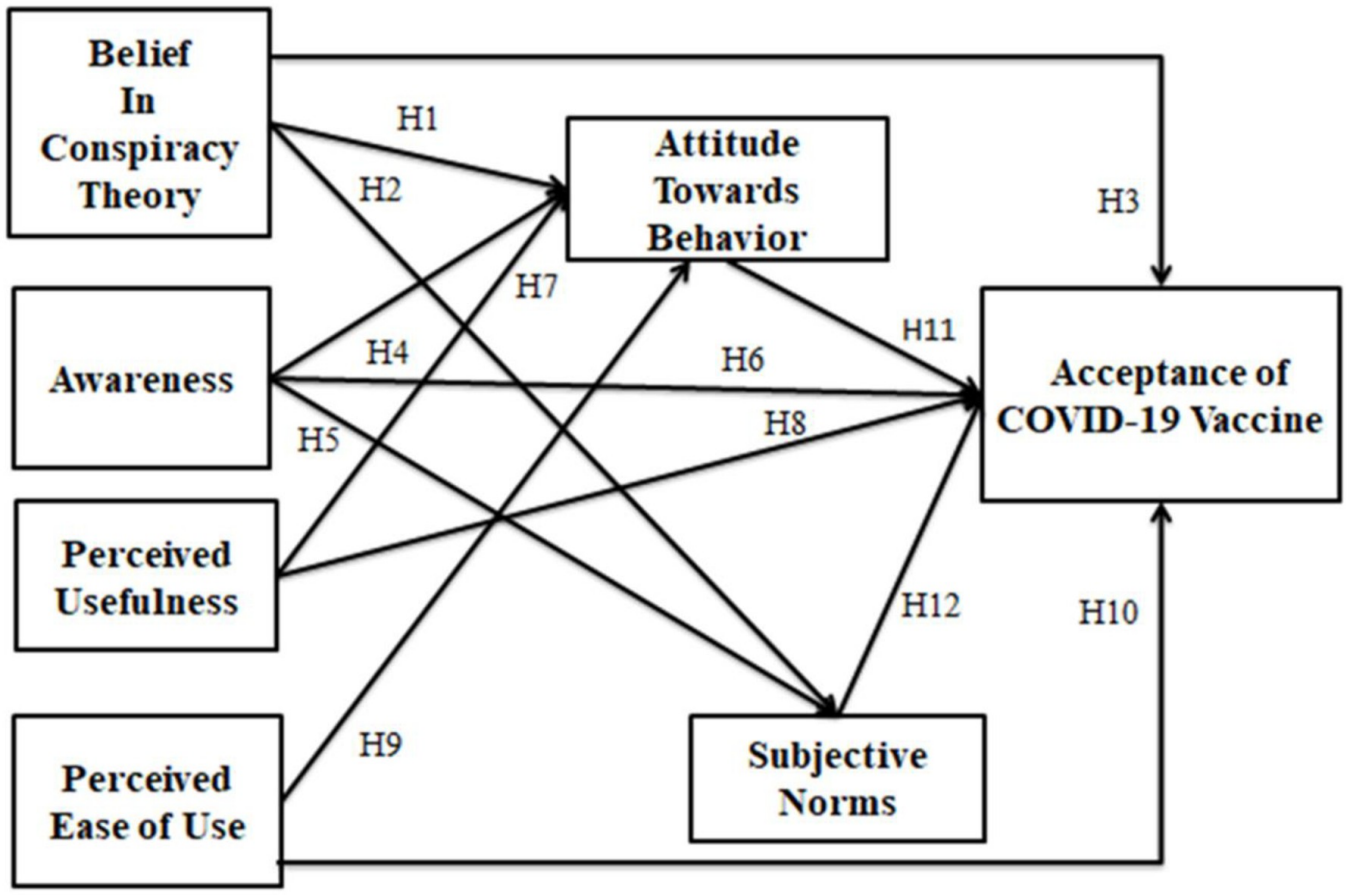
Conceptual model.

Fig 2 depicts the conceptual model of this study, while Table 1 summarizes the constructs and measurement items.

**Table 1:** Summary of constructs and measurement items.

Construct	Corresponding Items	Items Sources
**Belief in Conspiracy Theory (BC)**	BC1: I believe the government keeps many important secrets about the COVID-19 vaccine from the public. BC2: I believe the COVID-19 viruses and/or diseases have been deliberately disseminated to infect certain populations. BC3: I believe the rapid spread of COVID-19 viruses and/or diseases is the result of the deliberate, concealed efforts of some organization. BC4: I believe progress toward vaccination to cure COVID-19 is deliberately being hindered. BC5: I believe groups of scientists manipulate, fabricate, or suppress information about COVID-19 vaccine to deceive the public. BC6: I believe a lot of important information regarding vaccination is deliberately concealed from the public out of self-interest. BC7: I believe the government is involved in the murder of innocent citizens for not taking precautions against the COVID-19 pandemic and not making the vaccine widely available.	Brotherton, French, and Pickering [34]
**Awareness (AW)**	AW1: I follow the news and developments about the COVID-19 vaccine. AW2: I discuss the security issues of COVID-19 vaccine with friends and people around me. AW3: I read about the problems of taking the COVID-19 vaccine. AW4: I seek advice on taking the COVID-19 vaccine. AW5: I am aware of COVID-19 vaccination and its consequences.	Dinev and Hu [51]
**Perceived Usefulness (PU)**	PU1: I believe it is beneficial to protect myself from COVID-19 disease through vaccination. PU2: I believe protecting myself from COVID-19 disease enhances my effectiveness in continuing my work. PU3: I believe taking COVID-19 vaccine will enhance my effectiveness in my activities.	Davis [62]; Thurasamy et al. [74]
**Perceived Ease of Use (EU)**	EU1: The process of taking COVID-19 vaccine is clear and understandable. EU2: It would be easy for me to prevent COVID-19 thorough vaccination. EU3: It would be easy for me to be vaccinated.	Davis [62]; Thurasamy et al. [74]
**Attitude toward behavior (AB)**	AB1: I feel injecting COVID-19 Vaccine is a wise idea to protect against COVID-19. AB2: I feel injecting COVID-19 vaccine is a good idea. AB3: I like to use vaccine to protect against COVID-19 disease.	Thurasamy et al. [74]
**Subjective Norm (SN)**	SN1: Most people who are important to me would think that injecting COVID-19 vaccine is a wise idea. SN2: Most people who are important to me would think I should take the COVID-19 vaccine. SN3: My family who are important to me would think that taking the COVID-19 vaccine is a wise idea. SN4: My family who are important to me would think that taking COVID-19 vaccine is a good idea. SN5: My family who are important to me would think I should immunize through COVID-19 vaccine.	Thurasamy et al. [74]
**Acceptance of COVID-19 vaccine (AV)**	AV1: I have high intention to be immunized with a COVID-19 vaccine. AV2: I take vaccine to protect myself from COVID-19 disease currently. AV3: I would recommend the COVID-19 vaccine to my friends and colleagues to get protection from COVID-19 disease. AV4: Immunizing through COVID-19 vaccine is a pleasant experience for me.	Davis et al. [64]; Taylor and Todd [66]

## 4. Materials and methods

We examined COVID-19 vaccine acceptance using a structured questionnaire. The survey questionnaire was answered using a five-point Likert scale, with 5 indicating strong agreement and 1 indicating strong disagreement. The model was evaluated using PLS-SEM. This study aims to predict the key target construct and test new hypotheses. We used the PLS-SEM technique to evaluate the model because it offers the required features. PLS-SEM has widely used to measure causal relations among indicators and to reveal pivotal connections between the latent constructs [75, 76]. With the innovations in PLS simulations, PLS is a fully-fledged SEM approach [77–79].

### 4.1 Measurements of constructs and items

The Belief in conspiracy theory (BC) scale about COVID-19 vaccine was measured with a seven-item scale adopted from Brotherton et al. [34]. The adopted scales relate mostly to conspiracy belief about diseases or vaccines. We measured aawareness (AW) with a five-item scale adopted from Dinev and Hu [51]. PU, PU, and AB are measured on a three-item scale; slightly modified based on the purpose of the current study and adopted from Davis [62] and Thurasamy et al. [74]. Subjective norm (SN) is measured on a five-item scale adopted from Thurasamy et al. [74]. Finally, we measured COVID-19 vaccine acceptance (AV) using a four-item scale slightly modified for our context inspired by Davis et al. [64] and Taylor and Todd [66]. All the items were measured on a 5-point Likert scale ranging from 1 = strongly disagree, 3 = neutral, and 5 = strongly agree. The BC scale was reverse-coded. The questionnaire contains three parts. Part A contains demographic information; part 2 is on COVID-19 vaccine acceptance in terms of BC, AW, PU, PE, and the TRA. Finally, we asked some general questions about vaccines. We asked general questions in part 3 so that the respondents do not lose patience and answer the questions about the COVID-19 vaccine acceptance carefully.

### 4.2 Participants and data collection

The country was about to experience the third wave of COVID-19 at the time of this paper’s writing. The country-wide shutdown began with lockdowns. Almost all public universities are closed, and students are being housed in villages or in remote locations far from their universities. The best way to collect data was thus through an online survey. The ability to use an online survey is attributed to the proliferation of internet usage in Bangladesh as a result of increased government digitalization initiatives [80]. Using Google Forms, we created an online questionnaire and distributed the questionnaire to various universities’ Facebook pages. The most effective method was to ask teachers to distribute the questionnaire to different class and batch groups of students. Various Facebook Messenger and WhatsApp groups are being used during the pandemic to ensure proper communication with the students and to conduct online classes smoothly. Teachers distributed the survey to those groups, and the data were gathered from them between June 23rd and July 11th, 2021. We polled 351 respondents for their opinions. Participants in the survey were asked to provide informed consent. We kept a section in the Google form for respondents to express their open-ended opinions about the survey, and the majorities of respondents appreciated this research effort and see the survey as a medium to present their thoughts about the current pandemic and vaccination issues. 351 correct answers were used in this study, leaving out cases where the answers were incorrect or incomplete. In order to avoid overstating or exaggerating the study’s findings, all unmatched and incomplete cases were omitted. We include a mandatory step before submitting a response to the questionnaire in Google form to agree to the survey’s voluntary participation. The survey was conducted solely for research purposes and will not be used for commercial gain, with strict adherence to anonymity. Respondents were also assured that they could opt out of the study at any time. Table 2 contains the demographic information.

**Table 2:** Sample characteristics (n = 351).

Age	n (%)	University	n (%)	Residence	n (%)
19–22 years	221(62.96%)	Jagannath University, Dhaka	134 (38.18%)	Dhaka	193 (54.99%)
23–26 years	124(35.33%)	University of Dhaka, Dhaka	95 (27.07%)	Khulna	43 (12.25%)
27–30 years	5(1.42%)	Islamic University, Kushtia	47 (13.39%)	Chittagong	42 (11.97%)
Above 30 years	1 (0.28%)	Jatiya Kabi Kazi Nazrul Islam University, Mymensingh	37 (10.54%)	Mymensingh	32 (9.12%)
**Gender**	**n (%)**	Begum Rokeya University, Rangpur	18 (5.13%)	Rangpur	24 (6.84%)
Female	147 (41.88%)	Comilla University, Comilla	15 (4.27%)	Rajshahi	15 (4.27%)
Male	204 (58.12%)	Jahangirnagar University, Savar	4 (1.14%)	Sylhet	2 (0.57%)
**Level of Education**	**n (%)**	Hajee Mohammad Danesh Science and Technology University	1 (0.28%)	**-**	**-**
Graduation	317 (90.31%)	-	-	**-**	**-**
Post-Graduation	34 (9.69%)	-	-	**-**	**-**
**Grand Total**	**351**		**351**		**351**

#### 4. 2. 1. Ethics statement

Respondents provided informed consent and written statements about the voluntary participation, and their anonymity was strictly maintained. The study does not report a retrospective study of medical records or archived samples, and no minorities were reported.

**Fig 3:**
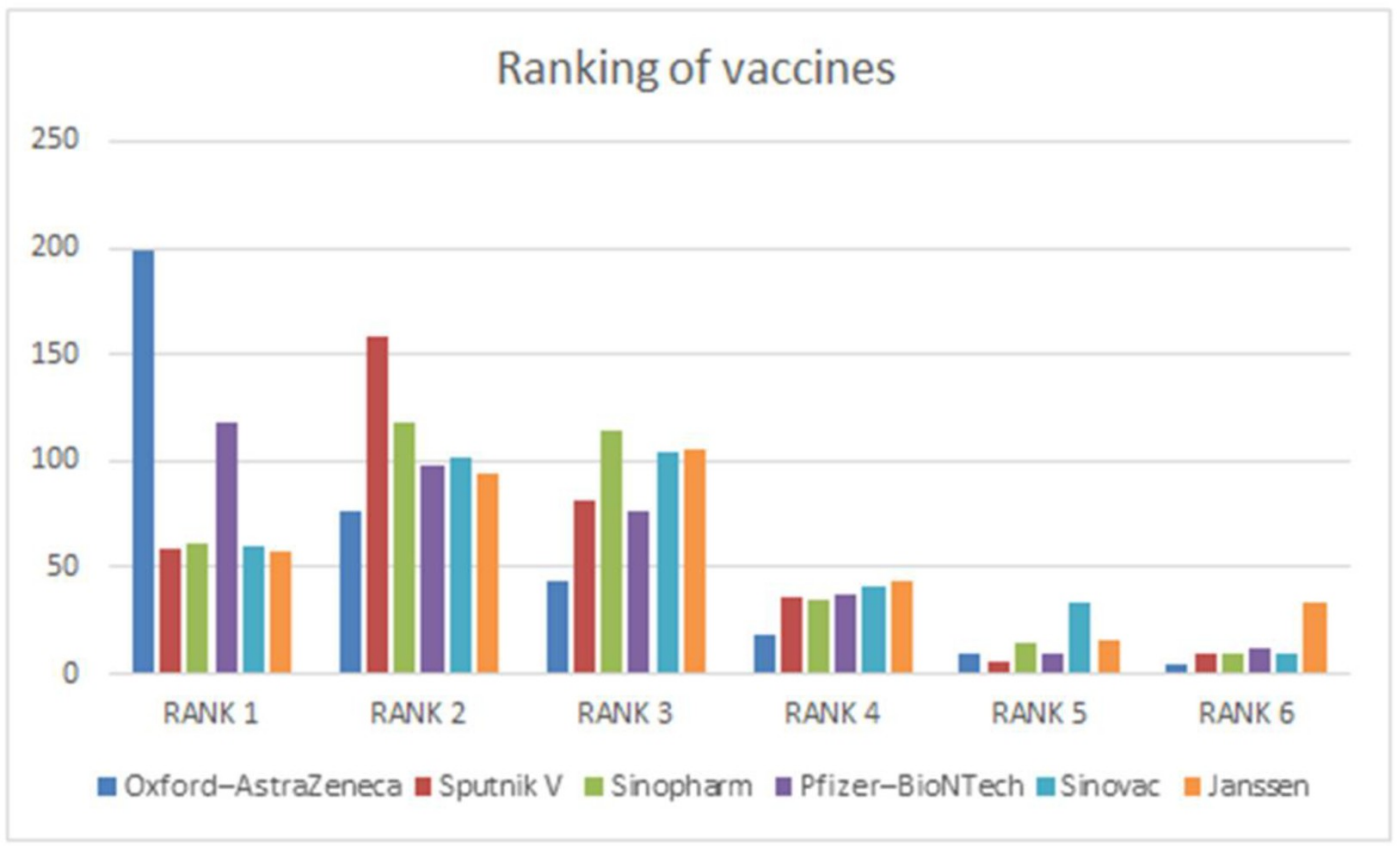
Vaccine ranking by public university students.

**Fig 4:**
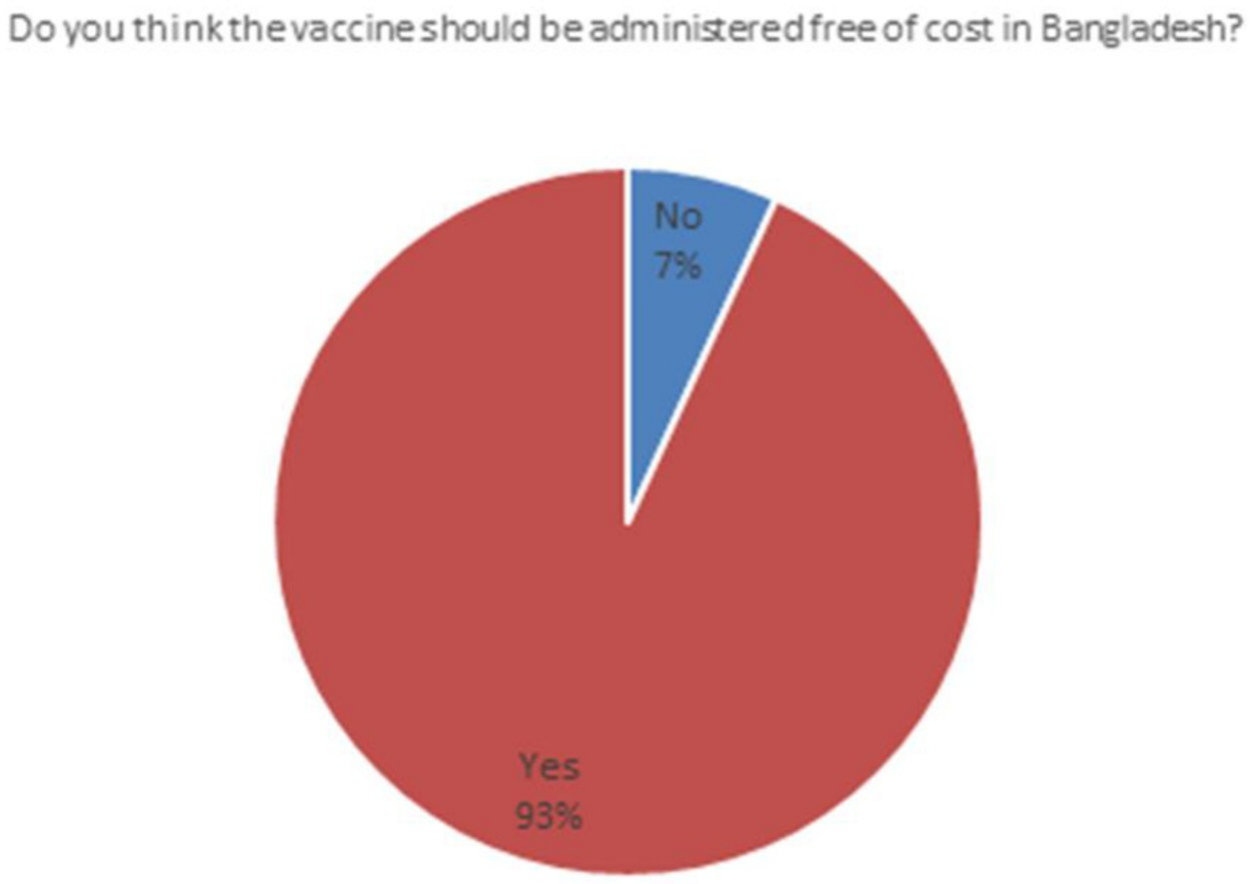
Vaccines should be administered free.

Further, we asked the respondents in part 3 of the questionnaire to rank the currently available and approved vaccines (as of 15th June 2021) in Bangladesh. Moderna Vaccine is not included in the rank as it was approved in Bangladesh on 29th June, 2021 (see Fig 3). We also inquired whether or not the vaccine would be provided free of charge (see Fig 4).

## 5. Results

### 5.1 Measurement model

As a variance-based SEM technique, the PLS path model involved two sets of linear equations: the measurement model and the structural model. The measurement model stipulates the interactions between a construct and its observed indicators, while the structural model stipulates the interactions between the constructs [77]. The reflective indicators are measured in terms of internal consistency, indicator reliability, convergent validity, and discriminant validity. To assess indicator reliability, indicator loadings should be higher than 0.70. For internal consistency, the Cronbach’s alpha value should be higher than 0.70, although in exploratory studies, 0.6 is acceptable. [81]. Composite reliability should be higher than 0.70 (in exploratory research, 0.60 to 0.70 is considered acceptable) [82]. Convergent validity is indicated by the average variance extracted (AVE), which should be higher than 0.50. For discriminant validity, the AVE of each latent construct should higher than the construct’s highest squared correlation with any other latent construct (Fornell–Larcker criterion) and an indicator’s loadings should be higher than all of its cross loadings [83]. We deleted BC2 and BC4 as they had considerably lower loadings; all other loadings were above 0.70. The reliability and convergent validity were quite satisfactory. Table 3 summarizes the assessment of the measurement model.

**Table 3:** Measurement model evaluation.

Construct	Item	Loading	Cronbach’s Alpha	rho_A	Composite Reliability	AVE
BC	BC1	0.804	0.784	0.755	0.78	0.518
	BC3	0.723				
	BC5	0.739				
	BC6	0.77				
	BC7	0.754				
AW	AW1	0.714	0.769	0.751	0.78	0.538
	AW2	0.71				
	AW3	0.7				
	AW4	0.765				
	AW5	0.754				
PU	PU1	0.8	0.704	0.799	0.83	0.621
	PU2	0.88				
	PU3	0.761				
EU	EU1	0.85	0.725	0.708	0.79	0.56
	EU2	0.68				
	EU3	0.86				
AB	AB1	0.851	0.84	0.849	0.903	0.757
	AB2	0.883				
	AB3	0.876				
SN	SN1	0.88	0.906	0.909	0.93	0.728
	SN2	0.82				
	SN3	0.84				
	SN4	0.91				
	SN5	0.9				
AV	AV1	0.813	0.829	0.829	0.887	0.662
	AV2	0.828				
	AV3	0.862				
	AV4	0.747				

### 5.2 Structural model evaluation

**Fig 5:**
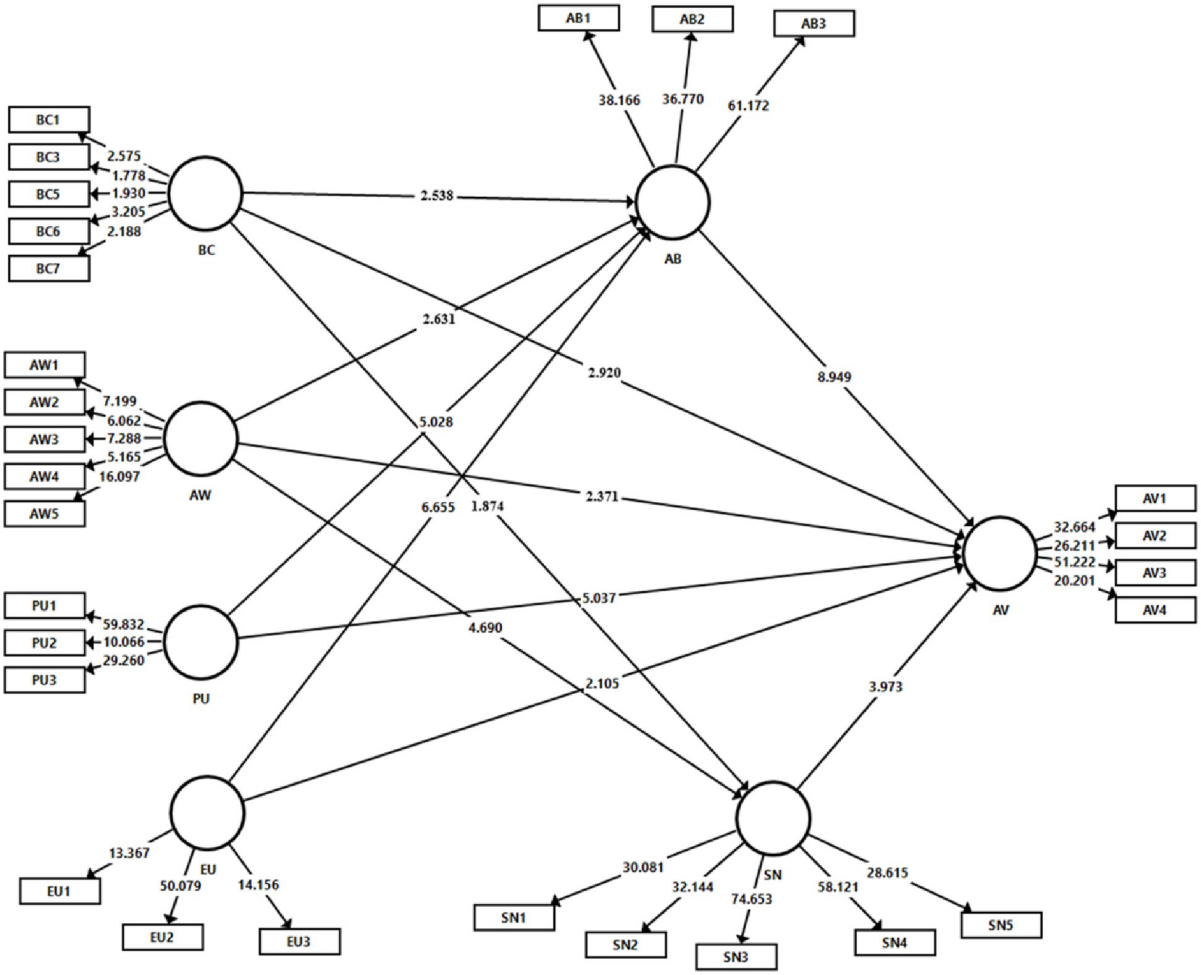
Empirical results for the structural model.

The bootstrapping technique (resampling = 5,000 minimum) was implemented to evaluate the statistical significance of the path coefficients [82]. In this step, we examined the proposed relationships between the exogenous and endogenous variables by the path coefficient (β) and t- statistics at a significance level of 0.1% (p< .001) and 5% (p< .05). As Table 4 reports, all postulated hypotheses for this study are confirmed and all were significant. We provide the structural model test results in Fig 5 and Table 4.

**Table 4:** Hypothesis testing results.

Hypothesis	Relationship	Standard Beta	Standard error	T Statistics	P Values
H1	BC→AB	-0.034	0.063	2.538	0
H2	BC→SN	-0.11	0.02	1.874	0.03
H3	BC→AV	-0.24	0.04	2.92	0.02
H4	AW→AB	0.04	0.06	2.631	0
H5	AW→SN	0.281	0.06	4.69	0
H6	AW→AV	0.021	0.035	2.371	0
H7	PU→AB	0.331	0.066	5.028	0
H8	PU→AV	0.208	0.04	5.037	0
H9	EU→AB	0.401	0.06	6.655	0
H10	EU→AV	0.087	0.041	2.105	0.036
H11	AB→AV	0.518	0.061	8.949	0
H12	SN→AV	0.19	0.048	3.973	0

### 5.3 Predictive relevance, R^2^ and Q^2^

The R^2^ indicates the variance explained in each of the endogenous constructs by the exogenous construct. It ranges from 0 to 1, with higher levels indicting more predictive accuracy. Hair et al. [82] advocated that R^2^ values of 0.25, 0.50, or 0.75 for dependent constructs in the structural model can be treated as weak, moderate, or strong, respectively. We find moderate R^2^ values for AB (0.412), SN (0.682), and AV (0.709), which are close to high, indicating that the proposed conceptual model explains an adequate portion of the variance in the COVID-19 vaccine acceptance.

The Q^2^ value provides another means to assess a model’s predictive accuracy [84, 85]. The Q^2^ value builds on the blindfolding procedure, which omits single points in the data matrix, imputes the omitted elements, and estimates the model parameters. Using these estimates as input, the blindfolding procedure predicts the omitted data points. This process is repeated until every data point has been omitted and the model re-estimated. The smaller the difference between the predicted and the original values, the greater the Q^2^ criterion and, thus, the model’s predictive accuracy and relevance. As a rule of thumb, Q^2^ values larger than zero for a particular endogenous construct indicate that the path model’s predictive accuracy is acceptable for this particular construct [86]. Our Q^2^ values for AB (0.299), SN (0.205), and AV (0.458) show good predictive relevance for the model of COVID-19 vaccine acceptance (see Table 5).

**Table 5:** R^2^ and Q^2^ values.

Construct	R^2^	Adjusted R^2^	Q^2^
AB	0.412	0.405	0.299
SN	0.682	0.680	0.205
AV	0.709	0.704	0.458

## 6. Discussion, contribution, limitation and conclusion

### 6.1 Discussion on the results

We aim to assess the factors that influence COVID-19 vaccine acceptance in the context of the pandemic. As 62.96% of the respondents were between the ages of 19 and 22, this study included a young sample with a university education. Females made up 41.88% of those polled. This survey was carried out at various universities throughout Bangladesh. The highest number of respondents came from two universities in Dhaka, the capital city, namely Jagannath University (38.18%) and the University of Dhaka (27.17%). We obtained no responses from Barisal out of the eight divisions of Bangladesh, and the highest response from Dhaka, 54.99%.

According to the study’s findings, BC undermines COVID-19 vaccine acceptance. The results for H1, H2, and H3 show that BC has a negative impact on individual attitudes and subjective norms toward immunization, which ultimately has a negative effect on COVID-19 vaccine acceptance. Individual awareness (AW), on the other hand, has a strong positive influence on COVID-19 vaccine acceptance. The results for H4, H5, and H6 show that AW has a positive impact on attitude, subjective norms, and COVID-19 vaccine acceptance. Furthermore, according to the findings for H7, H8, H9, and H10, PU and PE have a positive impact on attitude, subjective norms, and acceptance of immunization to protect against COVID-19. Individuals’ positive attitudes toward acceptance and constructive subjective norms have a positive impact on vaccine acceptance, as stated in H11 and H12.

Individual BC and rumors about vaccine formulation, development, distribution, and even effectiveness would have a negative impact on willingness to immunize. These negative thoughts would spread as normative behavior among their social groups, communities, and circles of belonging, potentially jeopardizing the overall success of the immunization program. Individual-level awareness can spread good thoughts among individuals and groups, leading to vaccination success and a reduction in overall infection. The perception that vaccination can protect against COVID-19 virus infection, severe illness, and death, can make students more willing to take the vaccine and to encourage others in their community to do so. The perceived ease of registering for and receiving vaccines without hassle or difficulty and receiving vaccines at no cost would encourage more students to get vaccinated.

### 6.2 Contribution of the study

Protective behaviors are critical in pandemic management, and vaccination may be a key protective behavior for COVID-19. Theoretically, we evaluate factors influencing COVID-19 vaccine acceptance by combining TRA with BC, individual awareness level, PU, and PE. Thurasamy et al. [74] decomposed the TRA and stated that PU and PE affect attitude. We find that BC affects attitude and subjective norms negatively and that awareness (AW), PU, and PE affect attitudes, which also affects acceptance positively.

Additionally, each of these objects can directly impact acceptance without affecting attitude or subjective norms. In this study, the path relationship of BC on AB is BC-AB, subjective norms BC-SN, and vaccine acceptance BC-AV is theoretically unique. BC has a negative impact on AB, SN, and AV. Individual-level awareness is related to AB, AW-AB, subjective norms AW-SN, and vaccine acceptance. Theoretically, AW- AV is also distinct here. AW has a negative impact on AB, SN, and AV. We also find that PU and PE have a positive impact on individual attitudes toward vaccination, which leads to acceptance. Both PU and PE have a positive effect on AB and AV.

This study makes an important contribution to policy development. Bangladesh is already considered a high-risk country, with the highest daily positive rate of 22.6% and an average positive rate of more than 9% from January to June 2021. Many of these people were asymptomatic and thus more dangerous to others in unknowingly spreading the virus. Hence, all people require vaccinations. Due to an increase in COVID-19 cases and a lack of vaccination, the plan to reopen universities has been postponed several times. This study aims to understand vaccine acceptance behavior among young people who attend public universities, whose health is a concern, and who will lead the nation in the future. The findings of this study will assist policymakers in their effort to improve vaccination success in a developing country context such as Bangladesh.

### 6.3 Limitations

This study employs an online questionnaire survey rather than a face-to-face administration. Students took part in the survey during the COVID-19 pandemic, when they were likely to be worried, frustrated, and living in uncertainty. This type of survey can be conducted with people from various segments of society, and comparisons can be made between their attitudes, beliefs, level of awareness, and acceptance of vaccines. In addition, we found a strong relationship between predictor variables and endogenous constructs. This model can be further tested by including mediating or moderating variables such as age, gender, religious beliefs, previous vaccination history, and so on, to gain a thorough understanding and exploration of the other influential factors to guide policymakers and save humanity from the COVID-19 pandemic.

### 6.4 Conclusion

This study investigated the factors influencing COVID-19 vaccine acceptance, which is a critical issue in the fight against the pandemic. The respondents were public university students studying various courses at the graduate and postgraduate levels, who will lead the nation in the future. Misinformation and conspiracy theories harmed vaccination programs in the past, including the recent COVID-19 outbreak. It is a cause for concern that if university students believe in conspiracy theories, they will spread faster and discourage people from taking vaccines. Additionally, if they are aware, have a positive attitude and beliefs, find vaccination useful in combating COVID-19, and can obtain the vaccine easily and free of charge, they can spread positivity, which will spread rapidly. According to our findings, BC has a negative impact, whereas individual awareness has a positive impact on individual attitude toward vaccination; group beliefs, which represent subjective norms; and actual acceptance of vaccines. The PU of the vaccines in combating COVID-19 disease, as well as the ease of the vaccination process, would have a positive impact on attitudes toward vaccination and ultimately vaccination acceptance.

The relevant authorities should focus on campaigns that could reduce misinformation and conspiracy surrounding the COVID-19 vaccine. Awareness programs are more important than ever, and individual-level awareness raising programs are required. Universities are tertiary-level educational institutes where students prepare for their future careers. A greater sense of awareness (free from conspiracy belief), usefulness of the vaccines, and ease of getting the vaccine would definitely help students immunize through the COVID-19 vaccine. If students are encouraged to believe that a vaccination certificate will be required to return to classes, continue their education, apply for full- or part-time jobs, apply for competitive government recruitment examinations, and that it may be necessary to travel abroad for higher education or a better job, they will gladly vaccinate themselves. Furthermore, easy, cost-free access to the vaccine will encourage them to get vaccinated. Because no medicine has yet been invented, vaccination is the only way to reduce infection, spread, serious illness, and deaths. Vaccination is the most effective way to protect individuals, families, and society as a whole. Families and communities will be able to gradually return to a more normal routine as more people are vaccinated.

## Supporting information

S1 Data (CSV)
